# Possible mediation of Cladocera species by a researcher's chest wader

**DOI:** 10.1016/j.heliyon.2023.e16725

**Published:** 2023-05-26

**Authors:** Arber Hajredini, Florent Demelezi, Imre Somlyai, István Grigorszky, Csaba Berta

**Affiliations:** a Department of Hydrobiology, University of Debrecen, Hungary; b Pál Juhász-Nagy Doctoral School of Biology and Environmental Sciences, University of Debrecen, Hungary; c Department of Water Management and Climate Adaptation, Institute of Environmental Sciences, Hungarian University of Agriculture and Life Sciences, Gödöllő, Hungary; d National Laboratory for Water Science and Water Security, University of Debrecen, Department of Hydrobiology, Debrecen, Hungary

**Keywords:** Cladocera, Mediation, Dispersal, Utilization type, Chest wader, Cleaning

## Abstract

Mediation of aquatic species has become an increasing problem for the last few decades. With the increasing commercial import, species’ direct or indirect spread can gain more space. There are several ways for them to land in their new home and spread through the country. Most of the aquatic species are spread by waterways, boats, vehicles, or even with the help of humans. Cladocerans have a good dispersal ability, thanks to their small size, additionally they possess good adaptation, and mechanisms to develop resting eggs. Benthic or littoral species can be mediated much more easily due to their living space, and with the help of human activities (e.g., scientists, anglers and people working in water bodies) they have a higher chance to colonize new habitats. Our goal was to explore if Cladocera species might be mediated by a scientist chest wader, while sampling in similar-sized, close-to-each other lakes, with different utilization. Most of the species were found in abandoned fishing lakes, followed by oxbow lakes (protected), and ultimately in intensively fished lakes. NMDS showed that samples from lakes with the same utilization are similar to each other. Differently utilized lakes can have various Cladocera species, even though they are closely related to each other. Based on the results, scientists can mediate species on their chest wader from lake to lake and may deteriorate the results. We recommend a necessary chest wader cleaning after every sampling process, especially when samples are taken from differently utilized lakes.

## Highlights

• The possibility of Cladocera species mediation by a chest wader was assessed for the first time.
• From the mediation point of view, utilization of an oxbow lake can be a decisive factor in the possible dispersion.
• Three main types of species dispersal were assessed based on the utilization type.
• Necessity of the equipment cleaning was confirmed, because of the high chance of species mediation.

## 1 Introduction

Areas with untouched conditions of conservation can be highly disturbed by human activities, which can deteriorate the preservation of these conditions [1]. Humans have migrated across the oceans for thousands of years [2,3]. Domesticated animals and plants were taken to help them in their new home, but unwillingly other organisms that were attached to their boats, clothes etc., were transported, representing the first long-distance dispersals by humans [4].

In the last half-century, commercial transport has increased significantly. This increase in transport has had an extraordinary effect on the dispersal of species, with or without intent. Research on the human role, in dispersal, has increased in the last decades [5]. Aquatic invertebrates’ natural dispersal vectors includes water connections [6,7], wind [8], amphibians [9], aquatic insects [10], waterfowl [11], and mammals [12].

Zooplankton are organisms that live in waterbodies (marine and freshwaters). They drift in the water, and most of them are microscopic, but some of the species can be seen by the naked eye. Their importance in the water food web is irreplaceable. Cladocerans or water fleas are an order of small crustaceans that feed on microscopic chunks of organic matter or algae. So far, over 650 species have been identified, with many more yet to be discovered [[13], [14], [15], [16]]. This study focuses on the human-mediated dispersal of Cladocerans because they are prevalent in almost all freshwater habitats [17], are fairly well-preserved when they die, and are used in several scientific studies to explain changes in environmental conditions of water bodies. Although they are widely distributed in water bodies around the world, some are more common in particular regions, and some may be threatened due to habitat destruction, pollution, or other factors. Different species have different habitat requirements, and it is possible for one species of Cladocera to outcompete other species in a given ecosystem [18], particularly due to their dominance in the study lake relative to other taxa, their high dispersal/adaptive capabilities, and if they have similar ecological niches or requirements. Additionally, it should be noted that these species are not alien or invasive taxa at the regional level; rather, they have ecological significance [19], and it is important to assess their abundance and diversity using sampling methods that do not introduce potential biases.

Zooplankton has high local dispersal capacities [20], but their ability to produce resting eggs increases their chances to disperse over large distances. Studies were made to see the role of different organisms on the long-distance dispersal of zooplankton e.g.- Coughlan et al. [21] studied the role of birds on zooplankton dispersal. Resting eggs can resist gut passage and can easily spread by fish and birds [11,21,22], which makes birds one of the ways for short or long-distance dispersal [23]. Birds shape community composition by direct dispersal and when there are changes in the water quality and productivity, they affect the post-dispersal of species [24].

Most of the research on the human role in the dispersal of aquatic invertebrates was focused on vectors related to ships because they represent an unintentional pathway for aquatic invertebrates’ dispersal worldwide [[25], [26], [27], [28]]. Human activities related to inland waters include maintenance and management of lakes, rivers, ponds, recreational activities, and research. Scientists visit aquatic habitats continuously for sampling or monitoring communities. The impact of these scientific activities on the dispersal of aquatic invertebrates have not been considered as it should in literature. The impact of footwear and motor vehicles has been considered by Waterkeyn et al. [12], who give evidence of frequent dispersal of aquatic invertebrates including Artemia, Cladocera, Ostracoda, Rotifera, Turbellaria, Nematoda, etc.

Most Cladocera species produce resting stages (ephippia) that are easily spread by water, wind, and other biological vectors [29,30]. It can withstand dehydration or freezing and can hatch in ideal conditions after long periods [[29], [30], [31]]. Their asexual reproduction method gives Cladocera advantages and makes them an easy distribution, but their fast-reproducing rate allows them to faster colonize the new aquatic environments. Some zooplankton species have successfully dispersed and invaded new aquatic environments over long distances, such as *Daphnia lumholtzi* (G.O. Sars 1885) [32], *Artemia franciscana* (Kellog 1906) [33] and *Daphnia pulex* (Leydig 1860) [34]. [35] said that an active resting egg bank consists only of the first few centimetres of the sediment. Human activities that interact with this sediment can easily take these resting eggs and affect the dispersal of freshwater invertebrates [5,36]. Another study by Valls et al. [37], demonstrated that the dispersal of species by scientific activities is possible. *Candona furtosae* (Teeter 1980) a species only found in Florida, has been found and distributed in Spain by sediment in scientists’ footwear, who have participated in an International Biogeography Society meeting.

Measures to prevent dispersal by human vectors should be taken to protect the preservation of protected lakes, otherwise, this vector could facilitate dispersal, and lead to a faster spread of alien species [37]. Evidence of Cladocera (*D. lumholtzi*) invading a lake by flowing in a supply pipeline 50 km from the receiving lake was shown by Havel and Shurin [26]. But reservoirs lacking upstream sources have been invaded as well, so there must be other ways of transport to disperse *D. lumholtzi* [32,38]. Waterkeyn et al. [12] were the first to study human-mediated dispersal by the mud attached to waterproof boots, and they described the dispersal possibility of this mechanism and its impacts on aquatic metacommunities. Cladocerans were found on 20% of the boot samples compared to Protozoans in 88%, Ostracods 40%, branchiopods (12%), bryozoans (8%), turbellarians (4%), etc.

Unnatural dispersal of aquatic species is a growing problem that is being exacerbated by a variety of factors, the most significant of which is human influence. Scientists' work, angler's work or recreational fishing, water company employees, and many other human activities – involving water – can have an impact on aquatic species distribution. The mediation problem we investigated encompasses a wide range of activities that, with or without consent, contribute to the problem's worsening.

Mediation is used in our research to describe the process in which scientists act as the intermediary party for Cladocera. This process is done unwillingly, but it does not imply that it will have a negative impact on the new environment. The term “human mediation” has been used in other studies in the field of environmental sciences. Merle et al. [39] described the importance of human mediation in species establishment: analysis of the alien flora of Estonia. Also, the work of Price et al. [40] in parasite mediation in ecological interactions describes the mediation as a term.

Based on the above-mentioned, we can state that all scientific activities that are related to inland water bodies, should be monitored to prevent aquatic species dispersal or mediation. Sampling processes that involve scientists having direct contact with water bodies, need to be controlled as well, by making sure that the equipment, clothes, chest waders and rubber boots are cleaned well before having contact with another water body to prevent mediation of aquatic invertebrates, and in our case disperse of Cladocera species. We aim to check the possibility of Cladocera species mediation by a scientist's chest wader during sampling and to finds out that different utilization can influence the mediation. We explored the possibility of our data can be false due to the lack of cleaning.

## 2 Materials and methods

**Fig. 1:**
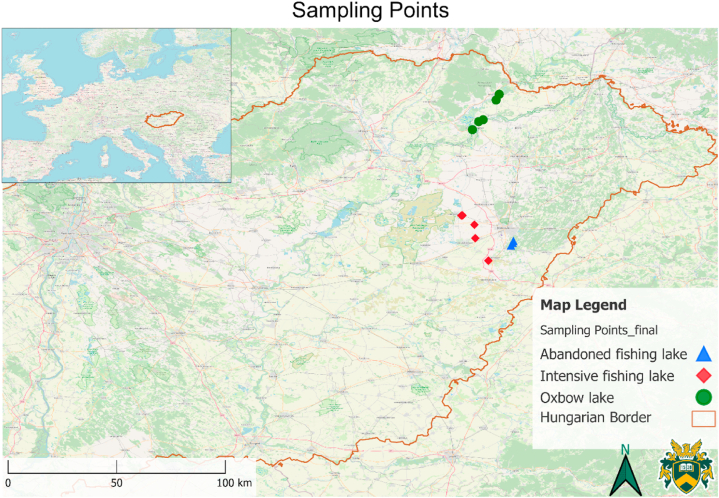
Location of the studied lakes with markings of different utilization and the border of Hungary.

The studied lakes are in the N and N-E Hungary (Fig. 1.) representing the micro-region of the Great Plain. During the experiment (2021 summer) we collected samples from 13 different standing waterbodies with three different types of utilization. The oxbow lakes (green marking) are functioning as a natural habitat, with naturally existing fish stock, but without fish installation and fishing activity. Lakes with red markings are utilized as fishing lakes with intensive fish installation (and intensive fishing), while lakes with blue markings are abandoned fishing lakes (Fig. 1). These lakes were once used intensively for fishing but dried out several times during the last decade and without a proper water supply, operators left them behind.

### 2.1 The mediation experiment

From each of the above-mentioned lakes, we collected samples with three repetitions. Before the sampling, all the researchers cleaned their chest wader with a mixture of soap and water and brushed it with a strong bristle brush. After the cleaning, the waders were rinsed well with de-ionized water. The researchers went into all the lakes, until the point where the water level reached the chest wader up most part, around 1 cm from the top section. The distance from the bank of the lakes is around 10 m. Based on previous samplings – this time was necessary to took a sample – so the researchers stayed exactly 10 min at the same place, and after the time limit, they left the lakes and immediately stepped into a clean plastic container, with a 10 L capacity. In the plastic container, they washed the chest waders down with de-ionized water, to avoid Cladocera species as contamination from a different source than the lake itself and used a soft bristle brush to gently remove all the particles attached to the wader. From the container, with the use of a 35 μm sized mesh, we sieved the samples into 50 mL centrifuge tubes and preserved them with 96% Patosolv alcohol. The process of cleaning the chest wader was repeated before and after at each sampling site.

### 2.2 Laboratorial work and processing of Cladocera samples

In the laboratory, we treated the samples with 100 mL of 10% KOH solution according to the standard method of Korhola and Rautio [41]. These mixtures were placed into plastic bakers and heated for 30 min at 70 °C in a Stuart SWB6D laboratorial water bath. After the treatment, all the samples were filtered through a 35 μm sieve to eliminate bigger particles. The prepared samples were also preserved with the above-mentioned 96% Patosolv alcohol to avoid fungal growth, and a few drops of Safranin-glycerine were added to dye the chitinized particles, to help the identification. We used an Olympus BX53 microscope and an Olympus DP26 digital camera for identification. During the identification process, we used 100 μL of samples per slide, and a total of 1 mL of samples were counted. Cladocera species were identified using the method of Frey [42], Błedzky and Rybak [43], and Gulyás and Forró [44].

### 2.3 Data analysis

During the statistical analysis, we used LogX transformation to normalise our data because our abundance data are strongly right-skewed. Based on Levene's test, the resulting p-value is smaller than 0.05 (p < 0.001), so we rejected the null hypothesis of equal variance and concluded that there is a variance between the assemblage differences. For the calculation of the diversity indices (Shannon-Wiener index and Simpson index), we used PAST ver. 3.17c (PAlaeontological STatistcis) software package [45]. Non-metric multidimensional scaling (NMDS) was used to represent the similarities and dissimilarities between the sampled lakes. To represent the pairwise dissimilarity between lakes in a low-dimensional space we used NMDS with the Bray-Curtis similarity index, based on the occurrence of the individual species. Map including the sampled lakes were generated by QGIS 3.16.10 programme.

## 3 Results

From the sampled 13 lakes, Cladocera specimens were found in all lakes except Kék víz. We found twenty-seven distinct species in the twelve lakes where Cladocerans were sampled. The Cladocera found includes the whole specimen, an ephippium, its remains or part of a remain. A high number of Cladocera species were found in oxbow lakes, most of these lakes were natural, protected water bodies, while the lowest number of species were found in intensively fished lakes, most of them manmade. There was a group of abandoned fishing lakes, which were not used for intensive fishing for the last 2–3 years, where the amount of Cladocera was remarkably high. In two oxbow lakes, the number of species was not at the same level compared to the others in this group, mostly because of long dry-out periods. From the intensively fished lakes, in Kék víz we did not find any Cladocera, while in Lake Vekeri-tó (VE) we found the highest amount of Cladocera, 1220 individuals. From oxbow lakes, the highest number of Cladocera was found in samples from Szabolcsi Holt-Tisza (SZ1) with 660 individuals, whereas the lowest number of Cladocera was found in Kis-Morotva tó (KMT) with 90 individuals (Table 1).

**Table 1:** The number of taxa and the number of individuals of Cladocera that were found in each of the lakes studied. Dominance, Simpson-, and Shannon-index are shown as well. Red color represents oxbow lakes, green color represents abandoned fishing lakes while blue color represents intensive fished lakes. Abbreviations are as following: TI (Tímári Holt-Tisza); SZO (Szögi Holt-Bodrog); KMT (Kis-Morotva tó); SZ1 (Szabolcsi Holt-Tisza); KBM (Keleti Holt-Bodrog); R (Rókás-tó); KTP (Kenu-pálya); VE (Vekeri-tó); L (Látóképi víztározó); KE (Kerek-erdei-tó); SZ2 (Szíki-tó) and S (Sáska-tó).

	TI	SZO	KMT	SZ1	KBM	R	KTP	VE	L	KE	SZ2	S
Taxa	17	17	6	14	9	16	21	22	11	22	3	3
Individuals	310	590	90	660	140	520	760	1220	150	1060	40	40
Dominance	0.0801	0.1106	0.2099	0.1405	0.1327	0.0880	0.0883	0.1037	0.1111	0.0943	0.3750	0.3750
Simpson	0.9199	0.8894	0.7901	0.8595	0.8673	0.9120	0.9117	0.8963	0.8889	0.9057	0.6250	0.6250
Shannon	2.6740	2.4750	1.6770	2.2520	2.1070	2.5710	2.6620	2.5400	2.3030	2.6290	1.0400	1.0400

From the total of 27 species, 24 distinct species were found in oxbow lakes, while 25 different species were found in abandoned fishing lakes and 24 different species were found in intensively fished lakes. *Monospilus dispar* (G. O. Sars, 1861) was found only in one oxbow lake while missing on the other types of lakes studied, although other species were found at least in 2 differently utilized lakes. Because the Kék víz did not have any species of Cladocera, we were excluded from our statistical data.

The number of distinct species of Cladocera found in all 12 lakes studied was 27, belonging to 4 different families: Bosminidae, Chydoridae, Daphniidae, and Sididae. Kerek-erdő (KE) and Vekeri-tó (VE) had the highest richness with 22 distinct species, while Sziki tó (SZ2) and Sáska-tó (S) had the lowest richness with 3 different species, respectively. The dominant species in our samples was *Bosmina longirostris* (O. F. Müller, 1776) found in 11 of 12 lakes. *Chydorus sphaericus* (O. F. Müller, 1785) was found in the highest numbers in all our samples with 1030 individuals while *M. dispar* was found only once in one of the oxbow lakes. The dominant species in oxbow lakes were *C. sphaericus* found in 4 of 5 lakes sampled with 210 individuals. In abandoned fishing lakes *C. sphaericus* was again the dominant species with 400 individuals found in all 3 lakes sampled. In the intensively fished lakes again *C. sphaericus* was the dominant species with 220 individuals in 3 of 5 lakes sampled.

The Simpson diversity index tells us that the highest average diversity is found in abandoned fishing lakes followed by oxbow lakes and the least diversity was found in intensively fished lakes. The lowest value of the Simpson-index was found in lakes S (Sáska-tó) and SZ2 (Szíki-tó), intensively fished lakes with 0.6250, while the highest value was found on lake TI (Tímári Holt-Tisza) oxbow lake, with a value of 0.9199 (Table 1).

Although the Shannon diversity index shows the same pattern. The highest diversity was found in abandoned fishing lakes and the lowest diversity was foundin intensively fished lakes. Oxbow lakes had moderate diversity. The lowest diversity was in lake S (Sáska-tó) and SZ2 (Szíki-tó) with a value of 1.0400 while the highest is found in lake TI (Tímári Holt-Tisza) with a value of 2.6740. The tables down below show the species of Cladocera found on each of the lakes sampled during our study (Table 2).

**Table 2:** Sampling sites with indication of the identified species and number of individuals (ind./L). Red color represents the oxbow lakes. Abbreviations are as following: TI (Tímári Holt-Tisza); SZO (Szögi Holt-Bodrog); KMT (Kis-Morotva tó); SZ1 (Szabolcsi Holt-Tisza) and KBM (Keleti Holt-Bodrog).

Species	TI	SZO	KMT	SZ1	KBM
*Acroperus harpae* (Baird, 1834)	2000	3000	1000	1000	
*Alona guttata* (G. O. Sars, 1862)		1000	2000	1000	5000
*Alona intermedia* (G. O. Sars, 1862)	1000	4000	1000		2000
*Alona quadrangularis* (O. F. Müller, 1776)	1000	1000			
*Alona rustica* (Scott, 1895)	1000				1000
*Alonella exigua* (Lilljeborg, 1853)	1000				
*Alonella nana* (Baird, 1850)	1000			1000	
*Bosmina longirostris* (O. F. Müller, 1776)	4000	9000		2000	11,000
*Camptocercus rectirostris* (Schoedler,1862)		1000			
*Chydorus gibbus* (G. O. Sars, 1891)	1000	1000		1000	8000
*Chydorus ovalis* (Kurz, 1874)	4000	2000	1000		6000
*Chydorus sphaericus* (O. F. Müller, 1785)	4000	6000		3000	18,000
*Diaphanosoma mongolianum* (Ueno, 1938)				1000	
*Eubosmina coregoni* (Baird, 1857)	2000			2000	3000
*Eubosmina longispina* (Leydig, 1860)		2000	1000	2000	2000
*Graptoleberis testudinaria* (Fischer, 1848)	2000	4000	3000		3000
*Leydigia acanthocercoides* (Fischer, 1854)		1000			
*Monospilus dispar* (G. O. Sars, 1861)		2000			
*Oxyurella tenuicaudis* (G. O. Sars, 1862)					1000
*Paralona pigra* (G. O. Sars, 1862)	1000	6000			1000
*Picripleuroxus laevis* (G. O. Sars, 1861)	2000	1300			3000
*Pleuroxus trigonellus* (O. F. Müller, 1776)	1000				
*Pleuroxus uncinatus* (Baird, 1850)	1000	2000			
*Pseudochydorus globosus* (Baird, 1843)	2000	1000			2000

Oxbow lakes were all-natural water bodies, they were protected, but lakes SZ1 (Szabolcsi Holt-Bodrog) and KMT (Kis-Morotva-tó) have a higher water level fluctuation, also they can dry out. We can see this in Table 2, Table 3. *C. sphaericus* was the most dominant species while *M. dispar* was found only once.

**Table 3:** Sampling sites with indication of the identified species and number of individuals (ind./L). Green color represents the abandoned fishing lakes, while blue color represents the intensively fished lakes. Abbreviations are as following: R (Rókás-tó); KTP (Kenu-pálya); VE (Vekeri-tó); L (Látóképi víztározó); KE (Kerek-erdei-tó); KV (Kék-víz); SZ2 (Szíki-tó) and S (Sáska-tó).

Species	R	KTP	VE	L	KE	KV	SZ2	S
*Acroperus harpae* (Baird, 1834)	2000	1000	1000		4000			
*Alona guttata* (G. O. Sars, 1862)	2000	2000	5000	1000	4000			
*Alona intermedia* (G. O. Sars, 1862)	7000	9000	9000		7000		2000	
*Alona quadrangularis* (O. F. Müller, 1776)	1000		2000		1000			
*Alona rectangula* (Sars, 1861)		2000			3000			
*Alona rustica* (Scott, 1895)	3000	1000	1000	2000	1000			
*Alonella excisa* (Fischer, 1854)			1000		1000			
*Alonella exigua* (Lilljeborg, 1853)		2000	13,000		9000			
*Alonella nana* (Baird, 1850)	4000		1000		1000			
*Bosmina longirostris* (O. F. Müller, 1776)	5000	7000	11,000	1000	11,000		1000	1000
*Camptocercus rectirostris* (Schoedler, 1862)	2000	1000	2000					
*Chydorus gibbus* (G. O. Sars, 1891)	2000	8000	17,000	2000	16,000			
*Chydorus ovalis* (Kurz, 1874)	5000	5000	2000	1000	3000			
*Chydorus sphaericus* (O. F. Müller, 1785)	6000	12,000	22,000	3000	18,000		1000	
*Diaphanosoma mongolianum* (Ueno, 1938)				1000				
*Eubosmina coregoni* (Baird, 1857)	1000	2000	1000					2000
*Eubosmina longispina* (Leydig, 1860)		4000		1000	3000			1000
*Graptoleberis testudinaria* (Fischer, 1848)	1000	5000	2000		2000			
*Leydigia acanthocercoides* (Fischer, 1854)		1000	1000		1000			
*Monospilus dispar* (G. O. Sars, 1861)								
*Oxyurella tenuicaudis* (G. O. Sars, 1862)	3000	1000	1000	1000	1000			
*Paralona pigra* (G. O. Sars, 1862)		1000	6000	1000	6000			
*Picripleuroxus laevis* (G. O. Sars, 1861)	7000	9000	17,000		11,000			
*Pleuroxus trigonellus* (O. F. Müller, 1776)		1000			1000			
*Pleuroxus uncinatus* (Baird, 1850)		1000	1000					
*Pseudochydorus globosus* (Baird, 1843)	1000	1000	5000	1000	1000			
*Simocephalus vetulus* (O. F. Müller, 1776)			1000		1000			

From all the abandoned and intensively fished lakes that were studied in this experiment, only lakes SZ2 (Szíki-tó) and KE (Kerek-erdei-tó) were natural water bodies, while the others were artificial, and all of them have water level fluctuation. As we can see in Table 3. *C. sphaericus* was the most dominant species in both types of lakes while *Pleuroxus trigonellus* (O. F. Müller, 1776) and *Simocephalus vetulus* (O.F. Müller, 1776) were the least found in abandoned fishing lakes, while in intensively fished lakes 7 species were found only once.

**Fig. 2:**
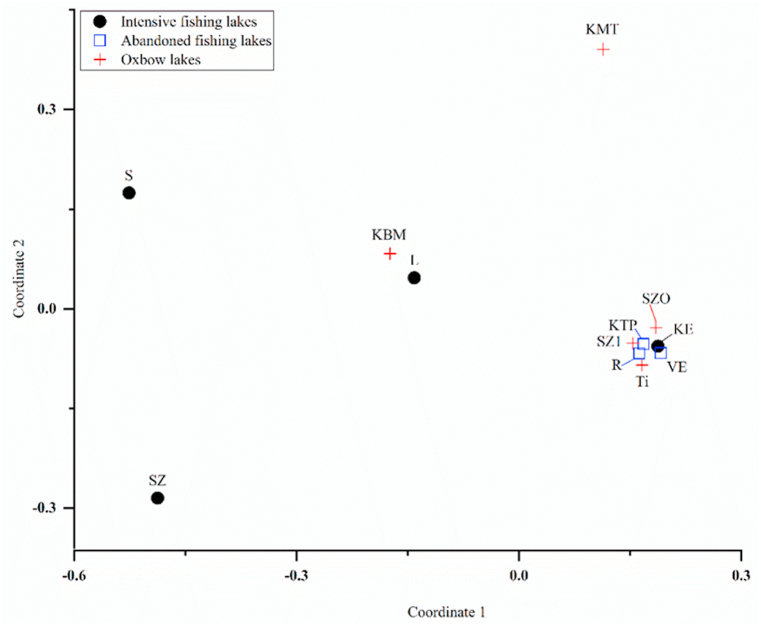
Non-metric multidimensional scaling (NMDS) with Bray-Curtis dissimilarity index. Analysis made on the Cladocera individuals grouped by the utilization types. Stress value is 0.09356. Red plus sign represents the oxbow lakes, black dots represent the intensively fished lakes, while empty squares represent the abandoned fishing lakes. Abbreviations are as following: TI (Tímári Holt-Tisza); SZO (Szögi Holt-Bodrog); KMT (Kis-Morotva tó); SZ1 (Szabolcsi Holt-Tisza); KBM (Keleti Holt-Bodrog); R (Rókás-tó); KTP (Kenu-pálya); VE (Vekeri-tó); L (Látóképi víztározó); KE (Kerek-erdei-tó); SZ2 (Szíki-tó) and S (Sáska-tó). (For interpretation of the references to color in this figure legend, the reader is referred to the Web version of this article.)

The NMDS shows the orientation of the lakes, based on Cladocera individual numbers from each lake taken from the chest waders after getting out from each lake (Fig. 2). Abandoned fishing lakes are grouped quite close to each other because the data from that group of lakes were very similar, while oxbow lakes were grouped close to each other except KMT and KBM. Intensively fished lakes are distributed in different areas, far from the other two groups except, lake KE. All five of the studied oxbow lakes are protected water bodies but they did not give us the same orientation. Lakes Ti, SZO, and SZ1 gave us equivalent results. Lake KMT and KBM are not in the same area as other oxbow lakes because they can easily dry out, while lake KBM can also be used for recreational fishing.

Abandoned fishing lakes were grouped in the same area, all three of them: KTP, R, and VE had a high number of Cladocera in the samples, and they are not used for fishing for the last three or more years. Lakes used for intensive fishing are the ones in which we found the lowest number of Cladocera specimens. Lakes S, SZ and L were positioned nearly the same way but not close to each other, while lake KE was orientated in the same way as abandoned fishing lakes, because of the high number of Cladocera found in the sample, due to natural fish stock without fish installation.

## 4 Discussion

There are a lot of natural vectors that can help the aquatic invertebrates be dispersed, and humans nowadays are one of the biggest factors influencing dispersal. It can make a willingly or unwillingly dispersal of aquatic organisms. Today there are thousands of cases where humans are the main reason for the dispersal of lots of distinct species. Over the last half-century, commercial transport has increased significantly, which had an extraordinary effect on the dispersal of species, with or without intent. Research on the human role, in dispersal, has increased in the last decades [5].

Every day, scientists around the world take samples from water bodies, for their daily or scientific research. Although taking samples on different water bodies in a short interval of time makes it somehow harder for changing or cleaning chest wader, this is a problem when the sampling sites differ from one another in terms of utilization type or other conditions. The number of aquatic species that can be attached to the chest wader of a scientist is high. Most of them can resist different conditions and stay attached easily to different objects, when they get in contact with new water bodies, they can thrive there without any problem.

Sometimes sampling process in limnology and paleolimnology approaches must be made by walking into the lake. This includes cases when renting or owning a boat is not reachable, when the water body is too shallow, or when sampling a high-mountain lake. There were many cases when the boats carry different aquatic organisms from one water body and disperse them to new aquatic habitats, even in the seas and oceans, different aquatic organisms are dispersed by boats and ships [4]. To prevent this, the small boats need to be cleaned after each sample-taking process. We wanted to know when the sampling is made by just walking into the water body and do the chest wader need to be cleaned before each sampling process. During walking into the lake until the sampling point is reached, our chest waders will be in constant contact with different aquatic organisms that can be attached to them and stay there for a period. Most of our Cladocera found during our study were remains, while we also found ephippia and whole organisms. After they die, Cladoceran remains are deposited in the sediment, during the sample-taking process the sediment is attached to the boots. The number of subfossil Cladocera is commonly higher than the number of contemporary Cladocera [46]. So, a good cleaning process is necessary to avoid false data in the samples. Attachment of different aquatic organisms found on the boots of scientists and then dispersal of this organism over long distances was described by Valls et al. [37]. When *Candona furtosae* (Teeter 1980) species, found only in Florida, was found hatched in Spain, from sediment by the scientists participating in an international biogeography meeting.

In this study, we tried to elaborate on the effect of scientist chest wader on the dispersal of Cladocera species. For the experiments we used three different utilization types of lakes: oxbow lakes, intensively fished lakes, and abandoned fishing lakes. Seven of the lakes, Tímári Holt-Tisza, Szögi Holt-bodrog, Kis-Morotva-tó, Szabolcsi Holt-Tisza, Keleti Holt-Bodrog, Kerek-erdei-tó and Szíki-tó, are natural water bodies while the others are man-made lakes. We wanted to know if the results depend on the utilization type of the lake, as well as if data can deteriorate if the chest waders are not cleaned between sampling processes and by this if scientists can have a role in the dispersal of Cladocera from lake to lake. In 12 of the 13 lakes sampled, we found at least one species of Cladocera, although only one our sample from lake Kék-Víz we could not find any species of Cladocera. The biggest factor of this Cladocera species missing is the high periods of drying out of this lake, which is accompanied by intensive fishing during periods of abundant water. Cladocera composition and density can rapidly change with the change in environmental conditions or climate change [47].

In oxbow lakes, we found a high diversity of species and a high number of Cladocera, because the conditions on these water bodies are favourable, and they have very small or no anthropogenic activities which explains our results. We also found species that were not found in other utilization types. High numbers of Cladocera species were found also in abandoned fishing lakes, with no fish introduction and low anthropogenic activities during the last years, so the Cladocera species had time to thrive. Many researchers found that zooplankton biomass will increase when fish biomass is decreased [[48], [49], [50]]. However in the investigated lakes that have a constant fish introduction, use and high human activities in intensively fished lakes the number and species stock of Cladocera in them were low. Boersma et al. [51] explained the great influence of fish on Cladocera species distribution. Species of contemporary Cladocera decrease in number because of fish predation as suggested by Berta et al. [52]. Apart from lake KE had quite a high amount of Cladocera found in the samples compared to other intensively fished lakes, even though is used for intensive fishing, there is no fishing introduced in this water body. The number of Cladocera species found in Hungarian water bodies is 98. We found 27 of them which is around 27.5% of all Cladocera species of Hungarian water bodies. *C. sphaericus* was the species that was found in the highest numbers in all 3 types of lake utilization. It is a species that can be found in a wide range of water bodies and ecosystems. It is also resistant to harsh environmental circumstances, with pH ranging from 3.7 to 9.9 in the finding sites and conductivity ranging from 0.4 to 957 mS/m, i.e., brackish water [[53], [54], [55], [56]]. Whereas *M. dispar* was found in one of the oxbow lakes only once, by reason that it was protected by water body. It can be found from near sea level to 663 m above sea level. The species is absent from the smallest ponds (less than 1 ha), although it is found in 15% of lakes larger than 1000 ha. It is a fairly acid-sensitive species that can be found in both vegetative and stony substrates. The discovered sites had conductivity ranging from 1.5 mS/m to 53 mS/m [[55], [56], [57]]. In this case, if we take the sample from this protected lake where *M. dispar* is usually found, and then we go to take sample to another lake which has a different characteristic where *M. dispar* normally cannot be found, there is a high possibility that we produce false data, because we may have carried the species on our chest wader, which is not properly cleaned.

Abandoned fishing lakes had higher values on average than oxbow lakes, while intensive fishing lakes had a low value on average, according to Simpsons and Shannon diversity indexes. The high diversity of abandoned fishing lakes is derived from the lack of fish introduction and low anthropogenic pressure on these lakes. Predation has the biggest impact on zooplankton communities compared to other biotic and abiotic factors [58]. In our survey, it was clear that during sampling in lakes, Cladocera as well as a big number of other aquatic organisms can easily be attached to our chest wader. Similar results were given by other studies on aquatic invertebrates’ dispersal [12,59].

Our study showed that when you go sampling on the same day to several water bodies, which of course differs in terms of utilization and protection from anthropogenic activities, a considered amount of Cladocera going to attach to our chest wader in each of the lakes. If we do not clean them after each sampling, there is a high chance that this can deteriorate our data and gives us false data on the species stock.

## 5 Conclusion

The number of Cladocera species in our samples was different among the utilization types. There have been distinct species of Cladocera found in the differently utilized lake, so the species stock varies based on the type of lake utilization. The sampling process needs to be performed with a clean chest wader to prevent mediation of Cladocera species and remains from one lake to another. Our data showed that scientists can disperse Cladocera with their chest wader, so a good cleaning process is a must-have before sampling, to get more accurate data and prevent deterioration of the results. To avoid the spread of aquatic species from one water type to another, we propose cleaning chest waders, scientists' clothes, anglers's clothes, and other human activity equipment used in natural water bodies.

**Fig. 3:**
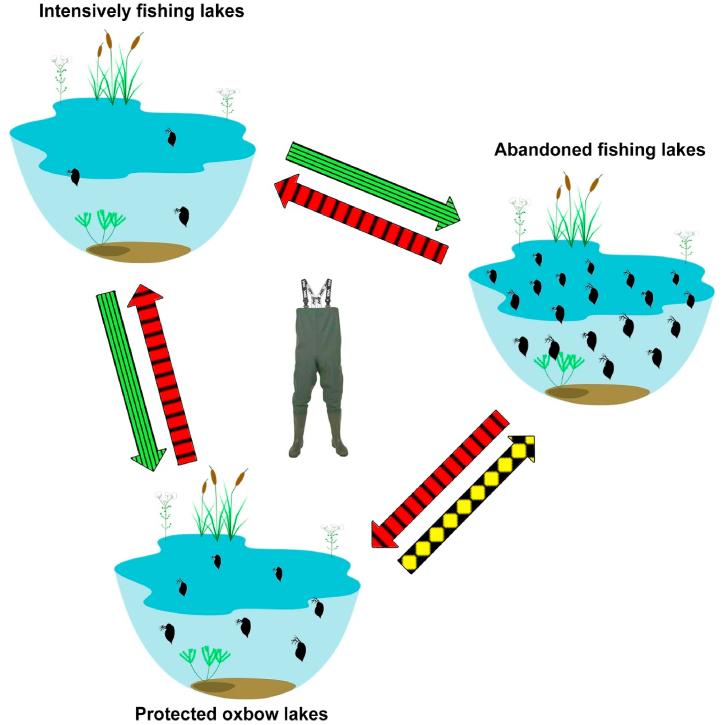
Schematic graph of the investigated differently utilized lakes with indication of the possible mediation routes. The vertically striped, red arrow indicates major possibility of species mediation. The horizontally striped, green arrow indicates a minor possibility of species mediation. The checkered, yellow color indicates moderate possibility of species mediation. (For interpretation of the references to color in this figure legend, the reader is referred to the Web version of this article.)

When there are conditions and possibilities, it is necessary to clean the chest wader. We devised a figure to prevent deterioration when sampling in lakes with varying utilization (Fig. 3). When we sample intensively fished lakes first, we have the least chance of deteriorating our sample, especially if we go to other utilization type without cleaning our chest wader, therefore cleaning is recommended in this case. If we sample in an abandoned fishing lake, we must clean our chest waders before moving into a differently utilized lake, otherwise, our samples will deteriorate. Cleaning is recommended in case when after sampling an oxbow lake we go to an intensively fished lake type, whereas cleaning is required if we go to an abandoned fishing lake to prevent deterioration. The green arrow (Fig. 3) indicates that cleaning is recommended, the yellow arrow indicates that cleaning should be done, and the red arrow indicates that cleaning of the chest wader is required.

## Author contribution statement

Arber Hajredini: Performed the experiments; Wrote the paper. Florent Demelezi; Imre Somlyai: Analyzed and interpreted the data. István Grigorszky: Conceived and designed the experiments; Contributed reagents, materials, analysis tools or data. Csaba Berta: Conceived and designed the experiments; Performed the experiments; Wrote the paper.

## Data availability statement

Data will be made available on request.

## Declaration of competing interest

The authors declare no conflict of interest.
